# Assessment of volumetric changes after regenerative endodontic procedures using semiautomated and 3D U-NET automated CBCT segmentation: a retrospective cohort study

**DOI:** 10.1186/s12903-025-06826-1

**Published:** 2025-09-16

**Authors:** M. Baraka, N. El-Kateb, M. Gamal, A. H. Elwan, P. Sharaf, L. Cevidanes, M. Torki, R. El Backly

**Affiliations:** 1 Pediatric Dentistry and Dental Public Health Department, Faculty of Dentistry, Alexandria University, Champollion St., El Azareta, Alexandria, Egypt; 2 Endodontics, Conservative Dentistry Department, Faculty of Dentistry, Alexandria University, Champollion St., El Azareta, Alexandria, Egypt; 3 Department of Computer and Systems Engineering. Faculty of Engineering, Alexandria University, Bab Sharqi WA Wabour Al Meyah, Al Attarin, Alexandria, Egypt; 4 Conservative Dentistry Department, Faculty of Dentistry, Pharos University, Canal El Mahmoudia Street, Beside, Green Plaza, Alexandria, Egypt; 5 Department of Orthodontics and Pediatric Dentistry, School of Dentistry, University of Michigan, 1011 N University Ave, Ann Arbor, MI 48104 USA; 6 Department of Computer and Systems Engineering, Faculty of Engineering, Alexandria University, Bab Sharqi WA Wabour Al Meyah, Al Attarin, Alexandria, Egypt; 7 Tissue Engineering Laboratories, Faculty of Dentistry, Alexandria University, Champollion St., El Azareta, Alexandria, Egypt

**Keywords:** Regenerative endodontic, Volumetric pulpal changes, Volumetric dentinal changes, Root length changes, 3D Unet model, Deep learning

## Abstract

**Background:**

This retrospective cohort study evaluated volumetric changes in dental pulp and root structure of necrotic teeth after regenerative endodontic procedures (REPs) compared to contralateral counterparts (CON) using semiautomated segmentation and a 3D UNet model.

**Methods:**

Data from 23 teeth with REPs and their CON were analyzed. Semiautomated segmentation was performed using ITK-SNAP and 3D Slicer CMF on scans taken before and 12 months post-treatment. Measurements included root length, volume changes (dentinal wall, intracanal calcification, and pulp) below the Biodentine plug. Automated pulpal segmentation utilized a 3D UNet model trained on 400 samples from the ToothFairy dataset, achieving a Dice Similarity Index of 0.76, Intersection over Union of 0.61, and Hausdorff distance of 2.15.

**Results:**

REP teeth showed no significant differences in root volume or dentinal changes compared to CON. REP significantly reduced pulp volume in mature teeth (-4.86 mm^3^ vs. -1.34 mm^3^, *p* = *0.05*), with lesser changes in immature teeth (-3.20 mm^3^ vs. -6.44 mm^3^, *p* = *0.12*). Significant root length differences (*p* = *0.04*) were observed between mature and immature teeth with REPs. The 3D UNet model and semi-automated method for volumetric pulp assessment showed excellent agreement (ICC = 0.92*, p* < *0.001*). Bland–Altman plots indicated good agreement between the two methods in measuring pulpal volumetric changes.

**Conclusions:**

REPs yield comparable findings to CON in root volume and dentinal wall changes, while significantly reduced pulp volume in mature teeth compared to CON. Distinct patterns of intracanal calcification and root length changes between mature and immature teeth were identified. A deep learning model can expedite post-REP pulp volume evaluations.

**Clinical implications:**

Understanding volumetric changes in dental pulp and root structure is vital for assessing REP success, facilitating clinical decision-making, and improving outcomes in regenerative endodontics.

**Supplementary Information:**

The online version contains supplementary material available at 10.1186/s12903-025-06826-1.

## Introduction

Regenerative endodontic procedures (REPs) are a widely applied treatment modality for management of necrotic immature permanent teeth and have been suggested as a potential alternative treatment for necrotic mature permanent teeth as well [1, 2]. According to the American Association of Endodontists (AAE) success of REPs has been categorized into primary, secondary, and tertiary goals [3, 4]. These are resolution of signs and symptoms and periapical healing, root lengthening and thickening, and restoration of tooth vitality hence allowing potential restoration of functional immune response and nociception [5, 6]. Indeed, survival and success rates following REPs in immature teeth have been documented to be as high as 92% and 84.3%, respectively when assessed based on the primary goal [7]. However, if root development is considered a critical criterion for success, it dropped to 60% [7]. While significant root development has been documented [8], only 27% of cases meet the clinically meaningful threshold of a 20% change in root dimensions [9]. These results highlight how unpredictable root development is following REPs. This is further complicated by the lack of reproducibility in the methods of analysis of root dimensional changes following REPs as well as the high incidence of intracanal calcification. Interestingly, both inconsistent root development and intracanal calcification have not been deemed as failures but rather as unsatisfactory outcomes following REPs [10].

While the evaluation of root dimensional changes post-REPs has progressed from linear measurements to assessing the radiographic root area (RRA), the majority of these analyses are conducted using 2D periapical radiographs, with only a few case reports or series providing exceptions [11, 12]. Such assessments are prone to inconsistencies and human error despite the use of positioning devices and special software which further contribute to why this outcome has been highly unpredictable until now [10]. Image distortion and the challenge of using reproducible landmarks, especially in growing young patients, further complicate assessments. Panoramic radiographs often overestimate pulp calcifications [11]. However, 3D imaging techniques, particularly cone beam computed tomography (CBCT) have reduced variability and allowed precise estimation of root development and pulp calcification following REPs [1]. Furthermore, the AAE has recommended the use of CBCT in REPs despite high radiation doses [3, 4].

Interestingly, continued root development, formation of intracanal calcific bridges and/or intracanal calcification may occur even in association with failed cases i.e., cases with persistent infection or recurring periapical lesions [13]. This can potentially complicate future treatment since rapid progressive intracanal calcification following REPs is best managed by immediate root canal treatment [7, 8]. A recent review by Nangia et al., 2021 [9], has recommended using reliable tools to assess volumetric pulpal changes before and after REPs, offering prognostic value for calcification rate and pattern. This helps researchers and clinicians determine necessary secondary interventions. Additionally, regeneratively treated teeth may need bleaching, orthodontic treatment, and restorations, the timing of which depends on achieving REP goals and the absence of negative outcomes. Furthermore, due to diverse REP treatment protocols, a reliable outcome assessment tool is crucial for steaming research and developing uniform guidelines for cell-homing and scaffold-based strategies mimicking native dentin/pulp tissue regeneration and understanding tissue healing mechanisms [9, 14].

Pulpal volume from CBCT scans allows for accurate and objective assessment of regenerative treatment outcomes using isotropic voxel in multiple orthogonal planes [12, 13]. Currently, dental pulp and root structure segmentation using Digital Imaging and Communications in Medicine (DICOM) files from CBCT scans can be performed manually which is time-consuming and demands a radiologist’s experience, or semiautomatically using label-specific correction tools resulting in rapid and more accurate measurements adjusting any small disconnected mistaken labels [14]. Semiautomated segmentation has been recognized as a reliable and valid method for radiographic volumetric assessment [15]. Validation of semiautomated segmentation and dental pulp volumetric assessment from CBCT after REPs by 3D Slicer CMF software demonstrated absolute agreement with real volumes measured by laser scanning and water displacement [16], and has also shown strong agreement with micro-CT [17]. ITK-SNAP 4.0 software also offers easy-to-use, cost-effective dependable tools for measuring radiographic volumes from CBCT scans [18].

Nevertheless, inaccurate semiautomated dental pulp segmentation can arise from threshold-based methods due to unclear margins in narrow pulp spaces [19]. Advances in deep learning models (DLMs) have shifted focus toward fully automated single-tooth segmentation using a two-phase approach: Region Proposal and Feature Pyramid Networks for bounding box extraction, followed by U-Net segmentation of hard tissue and pulp cavity [20]. Coarse-to-fine segmentation strategies with residual connections and attention mechanisms have been explored [21]. Multi-task feature learning has been utilized for instance segmentation, single tooth identification, and pulp region segmentation with Dental-Net and Pulp-Net [20]. DLMs, like the POSPADUNET model, which employs positional padding, serve as a baseline for pulp segmentation in CBCT [20–22], and have demonstrated superior performance in segmenting the inferior alveolar nerve and alveolar bone [23, 24]. DLMs can facilitate early diagnosis of failed cases post-REPs enabling timely interventions that improve clinical recommendations and evidence-based practices [25]. However, few DLMs have been used for pulp segmentation from CBCT scans, often trained on limited annotated data and teeth [20–22, 26–28]. Large datasets are essential for accurate DLMs, presenting a significant challenge and hindering research progress [24].

Semiautomated volumetric analysis of CBCT has mainly been used to study healing of periapical lesions after REPs [29]. The lack of published literature using semiautomated segmentation of dental pulp and root structure pre- and post-REPs in both immature and mature teeth using registered superimposed surface models from CBCT scans prompted our study. Hence, the first objective was to evaluate root length changes (primary outcome), volumetric changes in dental pulp, dentinal wall, and intracanal calcification after REPs, and compare them to physiological changes in the contralateral teeth (CON) after one year, using semiautomatically constructed superimposed 3D models from CBCT scans. The second objective was to compare the automated segmentation using the POSPADUNET model [23, 24], to semiautomated segmentation in assessing volumetric pulpal changes in mature teeth treated with REPs and assess the agreement of both techniques. The null hypothesis was that there would be no significant difference in volumetric and root lengths between REP and CON teeth measured by semiautomated segmentation, and the POSPADUNET model would demonstrate good agreement (ICC > 0.75) compared to semiautomated segmentation for pulp volume assessment.

## Materials and methods

This retrospective cohort study included records of patients with REPs from the Conservative Dentistry and Pediatric Dentistry Departments at the Faculty of Dentistry, Alexandria University, Egypt, from 2018–2022. Study protocols for mature and immature teeth were approved by the faculty’s research ethics committee (IRB NO: 00010556- IORG 0008839/22/1/2017 and 00010556- IORG 0008839/4/7/2018) and registered at clinicaltrials.gov (NCT04646538 and NCT04390854). The study was reported following STROBE and checklist for artificial intelligence in medical imaging (CLAIM) guidelines and GRRAS (*supplementary file*).

**Fig. 1:**
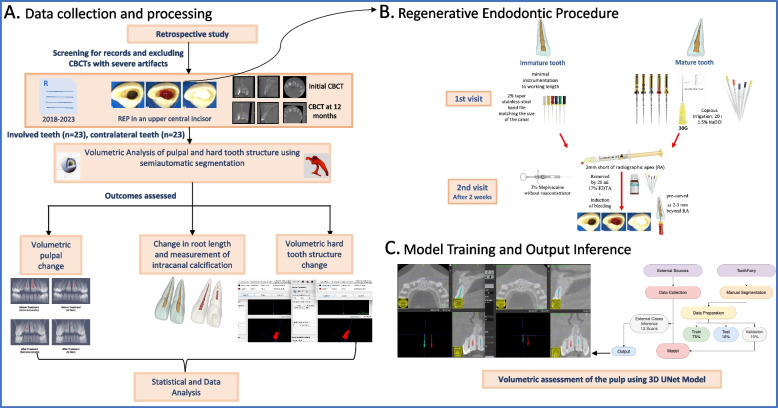
Overview of the study recruitment process, **A** Data collection and processing using semiautomated segmentation, **B** Regenerative Endodontic Procedure, **C** 3D UNet model

**Fig. 2:**
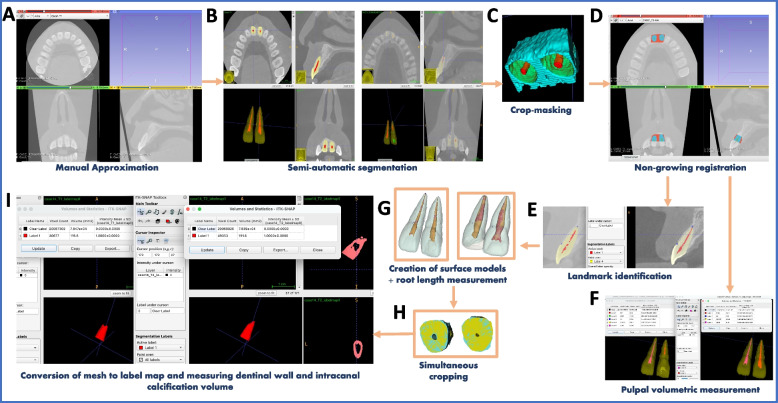
Representative graphical diagram of the image analysis steps; **A** Manual Approximation (Time 1&2), **B** Semiautomatic segmentation of the pulp and tooth structure of (Time 1&2), **C** Crop-masking after segmenting alveolar bone, **D** Non-growing registration (Registered Time 2 manually approximated scan and segmentation), **E** Simultaneous landmark identification (CEJ and RA), **F** Pulpal volumetric measurement (mm^3^), **G** Creation of surface models and measuring change in root length from CEJ to RA (mm), **H** Simultaneous cropping of the part of tooth structure apical to the biodentine restoration, **I** Conversion of mesh to label map and measuring dentinal wall and intracanal calcification volume (mm^3^)

The methodology of the study is depicted in Figs. 1 and 2. All procedures performed conformed with the 1964 Helsinki Declaration and its later amendments. Written informed consent was signed by the patient or the parents/guardian of the patient.

Sample size was calculated assuming a 95% confidence interval (CI) and 80% power to detect the difference in the changes in root length (primary outcome), volumetric dentinal changes, pulp volume between teeth treated by REPs as compared to their CON in mature and immature teeth. Meschi et al. [12] reported the mean (SD) change in root length in REP = 0.80 (0.98) and CON = 2.7 (2.5) mm, which rendered a sample size of 11 teeth. Meschi et al. [12] also reported the mean (SD) change in root hard tissue volume in REP = 5.2 (4.7) and CON = 25.2 (16.8) mm^3^, which rendered a sample size of 5 teeth. Sample size for the pulp volume changes was based on a pilot study, where the mean (SD) change in pulp volume in REP = −22.53 (15.17) and CON = −9.50 (5.56) mm^3^, which rendered a sample size of 8 teeth. The required sample size was calculated to be 44 teeth (highest number per group*number of groups (REP and CON)*number of subgroups (mature and immature teeth) = 11*2*2 = 44 teeth) which we increased to 46 (23 REP and 23 CON). G*power 3.1.9.7., MedCalc Statistical Software version 19.0.5 (MedCalc Software bvba, Ostend, Belgium; https://www.medcalc.org; 2019).

The study included records of male and female patients with traumatized immature central incisors up to Nolla stage 9 [30], aged 8–18 years, and traumatized mature central incisors, aged 25–40 years, all with periapical lesions on radiographs (*Digital radiograph software; 60kV/4mA, Owandy, Croissy-Beaubourg, France, ONE intraoral sensor; Croissy-Beaubourg, France*). They underwent REPs [31–33], with CBCT scans (*J.Morita R100 cone beam 3D imaging system, field of view (FOV) 50 mm* × *50 mm, isometric voxel size of 0.125 mm, 90 kVp tube voltage, 8 mA and 20 s exposure time) taken* at baseline and after 12 months. Records show that initially, all teeth responded negatively to percussion, palpation, and thermal and electric pulp testing. Periodontal examination showed physiologic mobility and normal probing depth (2–3 mm). After 12 months, all teeth exhibited clinical improvement and success outcomes were resolution of signs and symptoms; no pain, no sensitivity to percussion nor swelling, and reduction in the size of the periapical lesion radiographically using CBCT (supplementary file). DICOM–format CBCT images were anonymously extracted from the database (supplementary file, Fig. 1 (A-C)). Cases with poor quality CBCT scans or those showing severe distortion, due to artifacts, were not included. The current study only included successful regeneratively treated cases based on the resolution of clinical symptoms and periapical healing according to current guidelines.

### Semiautomated volumetric analysis of pulpal, dentinal wall changes, and intracanal calcification

CBCT images were analyzed by (MB, 15 years of experience in pediatric dentistry and CBCT analysis software) using ITK-SNAP (4.0, www.itksnap.org) and 3D Slicer CMF (v4.11.0, http://www.slicer.org) and steps shown in Fig. 2(A-I) [34, 35]. De-identified DICOM files were converted to nii.gz files using ITK-SNAP. Time 1 (T1) and time 2 (T2) scans were manually approximated (MA) using 3D Slicer CMF registration module taking upper incisors as the reference to apply and save matrix. Using ITK-SNAP, semiautomated segmentation of pulp and the root (dentinal wall and intracanal calcification) of REPs teeth and CON at T1 and MA-T2 scans were performed with different labels denoting different structures, including labial and lingual cortical bone helping with registration step through user-guided 3D active contour segmentation using level set methods [36]. Simultaneous mask cropping of both scans was done, occlusally at the cemento-enamel junction level (CEJ), and inferiorly at the apical third of the root of both REP and CON teeth by determining the radiographic terminus (RT) and the apical third is 3–5 mm coronal to RT. Using 3D Slicer CMF voxel base non-growing registration of T1 and MA-T2 scans and segmentations was done. In ITK-SNAP, root landmarks were labeled; CEJ and radiographic root apices (RA), in both T1 and registered MA-T2 scans, and volumetric pulpal changes were measured in both T1 and registered MA-T2 scans in (mm3) apical to the cervical Biodentine plug.

In 3D Slicer CMF, surface models for labeled T1 and registered MA-T2 segmentations were generated using Q3DC module. Fiducial marks were created using the labeled landmarks, to measure root lengths (CEJ-RA) in the superior-inferior plane and calculate change in root length (mm). Simultaneous clipping of T1 and T2 models of both REPs teeth and CON was performed, superiorly parallel to the top of the pulpal space (just below the Biodentine plug) for measurement of the dentinal wall and intracanal calcification volume apical to the restoration. Then, converting clipped surface model to segmentation was done (mesh to label map) to measure the root, dentinal wall and intracanal calcification volumes (mm3) in ITK-SNAP.

### Deep learning 3D UNet based model for automated volumetric pulpal analysis: proof of concept

A team consisting of two annotators led by MB, used the ITK-SNAP tool to segment the pulp of 400 scans from the publicly available ToothFairy dataset (443 scans) [link] [24], and obtained via CBCT (NewTom/NTVGiMK4, 3mA, 110 kV, 0.3 mm3 voxels) with volumes acquired at 0.3 mm intra-slice distance with a shape in the range from (148, 265, 312) to (178, 423, 463) for the Z, Y and X axes. The interval of voxel (grayscale) values was [−1000, 5264], crucial for accurately segmenting and analyzing the dental structures.

The inter-examiner and intra-examiner reliability were assessed by segmenting six scans, 3 weeks apart. The intraclass correlation coefficient (ICC) for the inter-examiner and intra-examiner reliability for volumetric pulpal assessments were (ICC = 0.986, 95% CI = 0.937, 0.997) and (ICC = 0.971, 95% CI = (0.933, 0.981), respectively.

The annotation task provided the gold standard segmented dataset (reference dataset) used to train a 3D UNet model (Figs. 1 and 2) to segment the pulp from CBCTs. POSPADUNET model was employed for automatic pulp segmentation to assess volumetric pulpal changes in mature teeth subjected to REPs and their CON dataset.

**Fig. 3:**
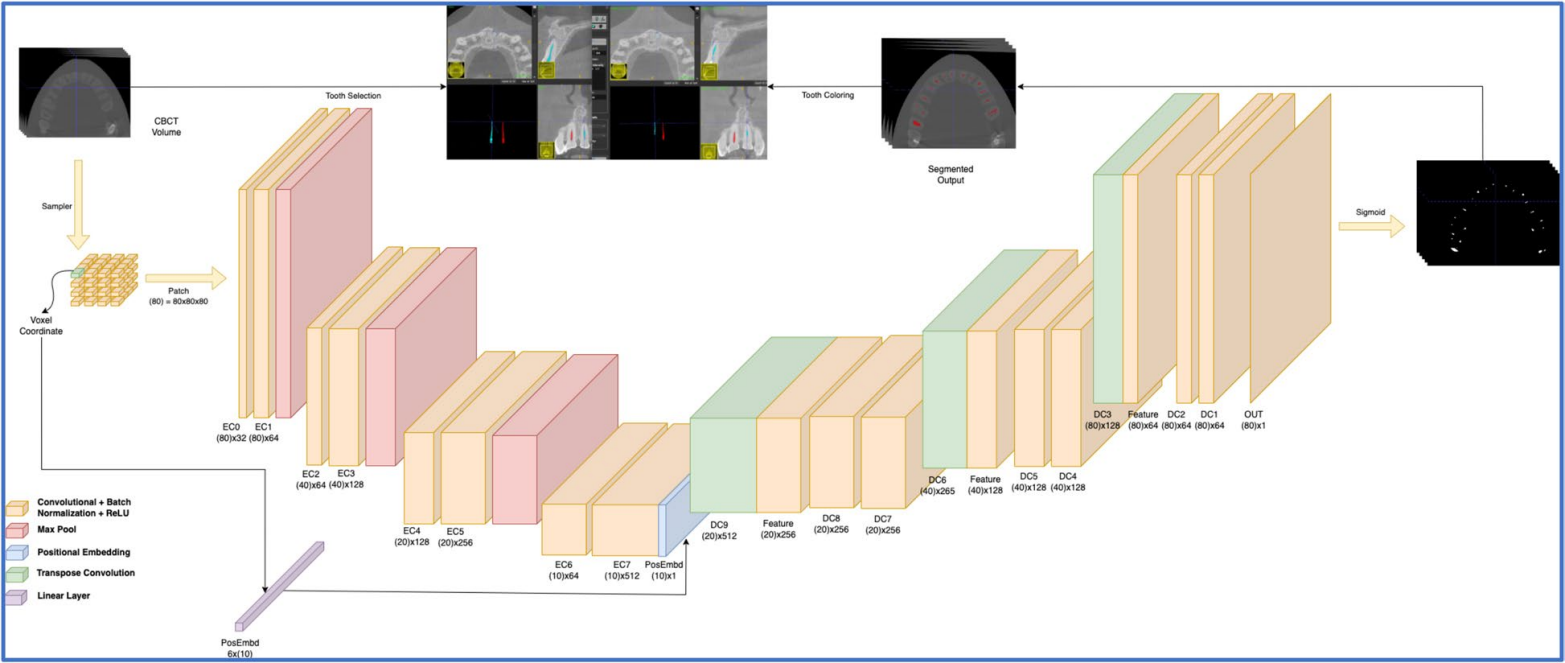
3D UNet model Architecture

Training and validation utilized 400 scans of ToothFairy dataset, averaging 14 teeth per scan, totaling 5600 mature teeth. Forty scans were reserved for testing. PyTorch and TorchIO were used for training the model on a Nvidia 4090-RTX GPU with 24 GB of RAM. Input sizes were padded and cropped into smaller patches (80,80,80), sampled via a Label Sampler. Stochastic Gradient Descent (SGD) with a learning rate of 0.1 and a plateau learning rate scheduler (Fig. 3) were used. The code is available in a private GitHub repository and Alexandria University repository upon request. Detailed hardware and software specifications are mentioned in the *supplementary file- Hardware and software specifications, and code/data sharing information* [24].

With performance metrics of 0.76 Dice Similarity Index, 0.61 Intersection over Union, and 2.15 Hausdorff distance, the 3D UNet model was externally validated qualitatively on twelve CBCT scans (six baseline, six after 12 months) of eight REP mature teeth (from our collected sample) to automatically segment the pulp and root canal, and measuring volumetric changes for each REP tooth and its CON using ITK-SNAP, comparing with semiautomated measurements (supplementary file, appendix Figs. 1−6). Time taken for automated (in seconds) and semiautomated segmentation (in minutes) of pulp space for REP and CON mature incisors was recorded using a digital stopwatch. Some noise outside the targeted pulp space being analyzed was manually removed; such noise would have been minimized with preprocessing of the scans. However, we did not perform any additional refinement of the automatically segmented pulp space to qualitatively validate the AI model.

Time taken for automated (in seconds) and semiautomated segmentation (in minutes) of pulp space of involved and contralateral incisors (mature teeth) from CBCT scans was recorded using a digital stopwatch.

### Statistical analysis

Data was analyzed using IBM SPSS Version 23 for Windows (SPSS Inc., Chicago, USA). The significance level was set at *p* < 0.05. Quantitative data were described by mean, standard deviation (SD), median, and interquartile range (IQR), while categorical data by frequency and percentage. Normality of the quantitative variables was tested using Shapiro–wilk test. Relationships between categorical data were assessed using Chi-square test. Associations between not-normally distributed variables were tested using Wilcoxon signed-rank or Mann–Whitney U tests. Intraclass correlation coefficient (ICC) determined reliability between semiautomated segmentation and 3D UNet model in measuring volumetric pulp changes with agreement shown by the Bland–Altman plot. 95% Confidence intervals (CI) were calculated. Microsoft Excel Windows365 and MedCalc2020 generated box-plots and Bland–Altman plots.

## Results

The analysis included records from a cohort of sixteen patients with twenty-three REPs in maxillary anterior teeth. Mature (*n* = 8) and immature teeth (*n* = 15) differed significantly in age (mean = 16.09 ± 8.85, *p* < *0.001*) and etiology (history of trauma or caries) (*p* < *0.001*), but not in gender *(p* = *0.18)* or diagnosis of pulp necrosis with asymptomatic apical periodontitis or chronic apical abscess *(p* = *0.31)* (*supplementary file-Appendix Table 1*).

**Fig. 4:**
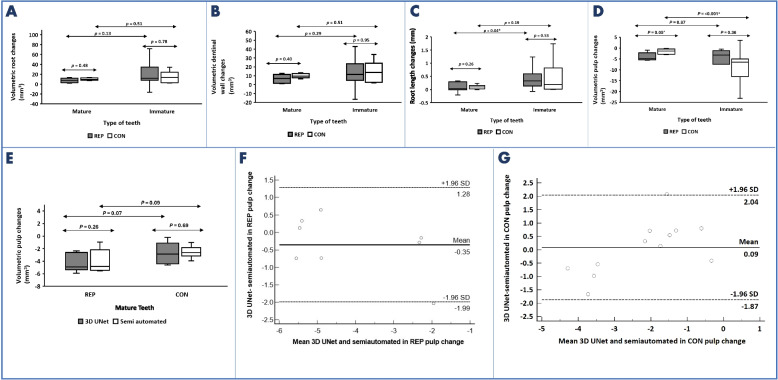
**A** Volumetric root changes in mature and immature teeth treated with regenerative endodontic procedures (REP) and their contralateral (CON) after 12 months (T2). **B** Volumetric dentinal wall changes in mature and immature teeth treated with REP and their CON after 12 months (T2). **C** Root length changes in mature and immature teeth treated with REP and their CON after 12 months (T2). **D** Volumetric pulp changes in mature and immature teeth treated with REP and their CON after 12 months (T2). **E** Difference between semiautomated and automated pulpal segmentation (3D UNet) in measuring volumetric pulpal changes in mature teeth treated with REP (*N* = 8) and their CON (*N* = 20) **F**\ Bland–Altman plot for the agreement between 3D UNet model (AI) and semiautomated segmentation (CBCT) in measuring volumetric pulp changes of mature teeth (both REP and CON). ^a^Wilcoxon signed-rank test, ^b^Mann-Whitney U test for the comparison between mature and immature teeth, SD standard deviation, *Statistically significant at *p* < 0.05

The median root volume change for REP was lower than for CON for both mature (7.40 mm^3^ vs. 9.65 mm^3^) and immature teeth (11.90 mm^3^ vs. 13.70 mm^3^), but the differences were not statistically significant *(p* = *0.48 and p* = *0.78, respectively)* (Fig. 4A, *supplementary file-Appendix Table 2*). Similarly, median dentinal wall changes for REP mature (6.86 mm^3^) and immature teeth (11.60 mm^3^) were lower than CON (9.65 mm^3^ and 13.70 mm^3^, for mature and immature teeth respectively), but these differences were also not significant *(p* = *0.40 and p* = *0.95)* (Fig. 4B, *supplementary file-Appendix Table 3*).

**Fig. 5:**
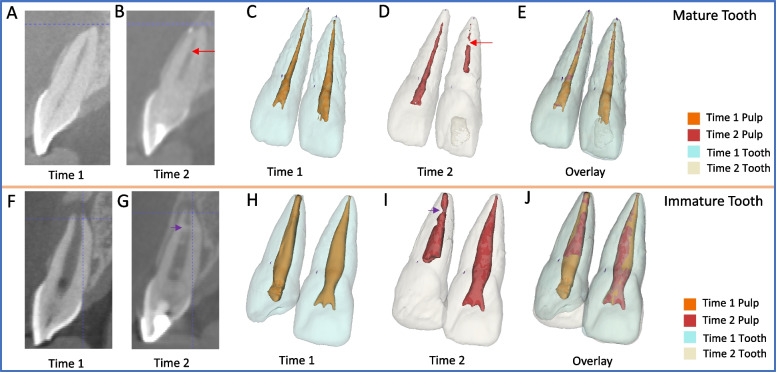
Representative radiographic images of two maxillary central incisors treated with REPs and their contralateral vital central incisors. **A**-**E** Mature case. **F**-**J** Immature case. **A**, **B**, **F** & **G**) Sagittal view of CBCT scans for both Time 1 and Time 2. **C** & **H** 3D surface models of the pulp, root canal, and hard tooth structure at Time1. **D** & **I** 3D surface models of the pulp, root canal, and hard tooth structure at Time 2. **E** & **J** Overlay of both Time 1 and Time 2 models. **B** & **D** Notice the red arrows showing calcific bridge formation in the revascularized tooth, both in the CBCT scans and the surface models of Time 2. **G** & **I** Notice the purple arrows showing the irregular dentinal wall thickening in the revascularized tooth

With respect to intracanal calcification volume, there was no significant difference between mature and immature teeth *(p* = *0.68)* (*supplementary file-Appendix Table 4)*. However, mature teeth with REP consistently displayed calcific bridges at an average distance of 4.34 (1.997 to 7.799) mm from the RA, while immature teeth with REP mainly exhibited irregular intracanal calcification, with only five cases showing a calcific bridge at an average distance of 4.95 (2.48–9.43) mm from the RA (Fig. 5A-J).

Regarding changes in root length in mature teeth, very minimal changes were observed; REP (0.03 mm) slightly lower than CON (0.10 mm) with no significant difference between both groups *(p* = *0.26)*. However, there was a greater change in REP immature teeth (0.33 mm) compared to CON (0.19 mm) but again, not statistically significant *(p* = *0.53)*. Immature REP teeth showed a significantly higher increase in root length than mature REP teeth *(p* = *0.04)* (Fig. 4C, *supplementary file-Appendix Table 5*).

As for volumetric pulpal changes in mature teeth, REP teeth showed a median change of −4.86 mm^3^, while CON teeth displayed a change of −1.34 mm^3^ with a significant difference between groups *(p* = *0.05).* While for immature teeth, less negative changes occurred in REP teeth (−3.20 mm^3^) compared to CON teeth (−6.44 mm^3^) with no significant difference *(p* = *0.12).* A significant difference, however, was found between immature and mature teeth only in the CON group *(p* < *0.001),* (Fig. 4D, *supplementary file-Appendix Table 6*).

When comparing volumetric pulp changes, in mature teeth, generated by the 3D UNet model to the semiautomated technique, there was no significant differences for both REP and CON (*p* = *0.26 and 0.69* respectively (Fig. 4E, *supplementary file-Appendix Table 7)*. Bland–Altman interpretation shows that there is good reliability between the 3D UNet model and semiautomated method in measuring volumetric pulp changes, ICC = 0.92, (*p* < 0.001). The Bland–Altman plot shows a bias = (−0.35 and 0.09) mm^3^ and limits of agreement = (−1.99, 1.28) and (−1.87, 2.04) in REP and CON, respectively (Fig. 4F-G). The mean time taken for semiautomated pulp segmentation was 21.0 ± 2.09 min while that for fully automated pulp segmentation was only 19.58 ± 5.28 s and this was significantly different (*p* < *0.001*).

## Discussion

This study aimed to optimize a semiautomated segmentation method to accurately assess changes in root length, pulpal volume, dentinal wall volume, and intracanal calcification post-REPs, and compare those to uninvolved teeth to minimize the influence of radiographic distortion and provide a true estimation of time-influenced changes occurring in young and mature individuals. Additionally, it aimed to establish a baseline for a deep learning-based pulp segmentation model as a prerequisite for developing a fully automated tool available for clinicians and researchers to detect volumetric pulpal changes post-REPs.

The semiautomated volumetric analysis used in our study, considered more accurate than manual contour tracing [16], involved registration and superimposition of overlaying surface models from CBCT scans taken 12 months apart to ensure consistency in landmark use and avoid overestimation or underestimation of changes in root length reported in prior studies [16, 35]. The present study distinguished between root volume changes as a whole, dentinal wall, root length and pulp volume changes to document more reproducible parameters. Although root development is measured cumulatively in terms of root thickening, lengthening and apical closure, all 3 parameters seldom co-exist following REPs hence the lack of predictable root development reported following REPs [37].

Threshold-based methods with unclear pulp space margins may lead to inaccurate semiautomated segmentation [19], prompting interest in DLMs for precise CBCT segmentation. Two-phase approaches, combining Region Proposal Network with Feature Pyramid Network from the panoramic view and U-Net models, have shown effective refined tooth and pulp segmentation [21]. Annotated CBCT datasets are crucial for DLM development and most of the research done used limited private datasets [20, 21, 38]. Notably, Pulpy3D Dataset stands out with 443 CBCT scans annotated for inferior alveolar nerve (IAN) and teeth pulp segmentation [24], facilitating POSPADUNET model training, validation, and testing. Further evaluation of the model on twelve scans of our current dataset with similar FOV was carried out. Our study uniquely measures longitudinal volumetric changes post-REP using DLM, contrasting single-time-point studies focused on forensic or non-clinical outcomes [20–22, 26–28].

In the current study, there was no statistically significant difference in root volume changes or dentinal wall changes between REP and CON teeth in both mature and immature teeth. In contrast, EzElDin et al. 2015 [17], reported significant hard tissue formation in teeth with REP overtime. Most published studies in immature teeth only compare baseline and last endpoint values excluding that seemingly normal contralateral teeth are also subject to changes with time. This may provide an over-estimation of clinically meaningful changes in immature teeth subjected to REPs [7, 9]. This disagreement with previous studies [8, 17], may also be due to the enhanced accuracy of simultaneous measurements from superimposed registered models of initial and 12-month scans in this study. The impact of analysis methods on inaccurate root development estimates post-REPs was noted by He et al.,2017 [35]. Furthermore, to our knowledge, no previous studies attempted to measure these volumetric changes in mature teeth after REPs.

Indeed, no significant differences existed in root length between each tooth and its contralateral in either group, although the median change in root length for the immature REP group was slightly more than the healthy contralateral tooth. It was also noteworthy that while contralateral tooth roots in the immature group showed increased length consistent with tooth maturation, the mature contralateral teeth also changed in length. The average change in root length in immature teeth with REPs was significantly more than that in mature teeth. These noted changes may not be very relevant as they also happen in healthy teeth over time [39]. It is also important to note that the etiology of pulp necrosis may have a profound negative impact on the outcome of REPs particularly in relation to root development and it may also influence the aggravated occurrence of intracanal calcification following REPs in both mature and immature necrotic permanent teeth. Most of the cases in this study had an etiology of trauma. Trauma has been correlated with outcomes such as unpredictable root development, root resorption and intracanal calcification and is a predictor of higher failure following REPs [40], This emphasizes the need for more reliable outcome-measuring methods for REPs taking into consideration normal physiological changes in healthy teeth. Additionally, variability in REP protocols, intracanal medicament choice, coronal plug material, and other factors may affect hard tissue formation [1].

Regarding intracanal calcification, our study findings indicate that while intracanal calcification volume is similar in mature and immature teeth, the organization of calcification differs significantly. Mature teeth with REPs tend to form consistent calcific bridges at a specific distance from the root apex, suggesting a more structured response. To our knowledge, this has not been previously documented in mature teeth. In contrast, immature teeth predominantly exhibited irregular calcifications, which may again be an influence of the traumatic insult that these teeth had received. A previous study of 3 cases showed that a calcific barrier consistently formed cervical to the coronal plug material [40]. However, the authors could not explain the reasons and suggested that several factors may result in different healing patterns following REPs.

Moreover, our study revealed that REPs significantly reduced the pulp volume in mature teeth compared to the healthy contralaterals. In contrast, immature teeth showed less reduction in pulp volume with REPs. This could be related to the type of stem/progenitor cells recruited during REPs. While immature teeth may still have preserved stem cells from the apical papilla which would contribute to the formation of a more physiologic “dentin/pulp-like tissue” [37], most of the cells recruited in mature teeth have probably higher cementogenic and osteogenic capacities leading to more reduction of pulp volume. Interestingly, immature teeth experienced a greater pulp volume reduction in the CON group compared to mature teeth. This may again reflect the etiology of trauma leading to more accentuated responses in immature versus mature teeth as the former would naturally have higher turnover rates.

Concerning the 3D UNet model use, our findings indicated that both the 3D UNet model and the semiautomated technique yielded comparable volumetric pulp changes, with no significant differences observed for either REP or CON teeth. The high intraclass correlation coefficient further confirms the reliability of both methods, suggesting they produced consistent measurements. Additionally, the Bland–Altman plot supported the good agreement between the two techniques. However, a notable difference in processing time was observed, with the semiautomated method taking an average of 21.0 ± 2.09 min, compared to just 19.58 ± 5.28 s for the fully automated approach. This highlights the efficiency of the automated method, making it a preferable option for pulp segmentation despite both methods demonstrating reliable results. This marked efficiency gain could substantially improve clinical workflows and research productivity.

Therefore, our study partially rejected the null hypothesis as there were significant differences between the average root length change in immature and mature REP teeth, pulp volume in mature REP teeth compared to healthy contralaterals as well as between pulp volumes of contralateral mature and immature teeth, and only the processing time between semiautomated and automated techniques.

Using neural networks for rapid, automated pulp space analysis could significantly impact clinical decision-making post-REPs. By providing objective, quantitative data on pulp volume changes and calcification in near real-time, the developed AI tool, designed for integration into existing CBCT software, will enable rapid assessment of pulp volume changes and intracanal calcification post-REPs, improving diagnostic accuracy. This swift detection of unfavorable outcomes will allow dentists to make timely decisions regarding retreatment necessity, optimize recall schedules for optimal timing of final restorations and ultimately enhance procedural outcomes while reducing costs associated with delayed or unnecessary interventions [2, 5–7].

The 3D U-Net model was initially trained and validated using a large dataset of 400 annotated CBCT scans (~ 5600 teeth), enabling generalization to CBCT scans with similar parameters acquired from different devices and populations. However, the small sample of REP cases for reliability testing and the retrospective design are study limitations. Furthermore, the 3D U-Net model could only be tested on the mature teeth dataset due to the differences in the resolution and exposure parameters of the CBCT scans taken for the immature teeth. However, we believe that in doing so this opens immense prospects for assessing outcomes of REP studies performed in mature teeth. This emphasizes the need to further train the model to take into consideration these heterogeneities in the future. Large-scale, prospective studies are necessary to confirm the findings’ generalizability across diverse patient populations, REP protocols, and CBCT acquisition parameters and to integrate clinical and imaging outcomes. Findings related to the comparison between the automated POSPADUNET model and semiautomated segmentation, as well as subgroup analyses of volumetric changes between mature and immature teeth, should be regarded as preliminary and hypothesis-generating due to the small sample size included in the current study, necessitating further validation in future research. Another limitation for the study, is the lack of ability of CBCT to distinguish between vital and non-vital tissue. Additionally, the relatively short follow-up period of 12 months for the REP cases is a limitation, and we recommend longer follow-up durations to better assess the long-term outcomes.

In conclusion, this study supports prior findings on successful outcomes of REPs for both mature and immature necrotic permanent teeth [1, 2]. Regenerative endodontic procedures resulted in comparable median changes in root volume and dentinal wall thickness compared to CON for both mature and immature teeth. Notably, REPs led to a significant reduction in pulp volume for mature teeth compared to CON, while immature teeth exhibited a less negative change in pulp volume with REP compared to CON. Additionally, a significant difference in pulp volume reduction was found between immature and mature teeth in the CON group. The study found distinct patterns of intracanal calcification and root length changes between mature and immature teeth. Importantly, the comparison of volumetric pulpal assessment using the 3D UNet model and semiautomated method demonstrated high reliability, with the automated method significantly reducing segmentation time, suggesting its potential for efficient clinical application.

## Supplementary Information

Supplementary Material 1.

## Data Availability

ToothFairy dataset is a publicly available dataset [link] (https://ditto.ing.unimore.it/toothfairy/?fbclid=IwAR2gRzMOyfsWnRUgscWV1Bhx1BW2BqhUsJs5s66vPH2cai0Cf_DqiftLzyw)] (443 scans dataset) [23]. The twelve CBCT scans are stored as a zipped file in the Alexandria University repository. The code is available in a private repository on GitHub as well as in the Alexandria University repository upon request from the corresponding author on GitHub as well as in the Alexandria University repository upon request from the corresponding author (M.B., email: marwa.baraka@alexu.edu.eg).

## Abbreviations

REPs: Regenerative endodontic procedures
AAE: American Association of Endodontists
CBCT: Cone beam computed tomography
DICOM: Digital Imaging and Communications in Medicine
DLMs: Deep learning models
CI: Confidence interval
CON: Contralateral
MA: Manually approximated
CEJ: Cemento-enamel junction level
RT: Radiographic terminus
RA: Radiographic root apices
