# Supplementary material for: Assessment of volumetric changes after regenerative endodontic procedures using semiautomated and 3D U-NET automated CBCT segmentation: a retrospective cohort study
Source: BMC Oral Health. 2025 Sep 16;25:1409. doi: 10.1186/s12903-025-06826-1 (PMC12442267; doi:10.1186/s12903-025-06826-1)
Supplement: Supplementary file 1 — Supplementary Material 1. [file 12903_2025_6826_MOESM1_ESM.docx]

STROBE Statement—Checklist of items that should be included in reports of ***cross-sectional studies***

|  | Item No | Recommendation | | Page |
| --- | --- | --- | --- | --- |
| **Title and abstract** | 1 | (*a*) Indicate the study’s design with a commonly used term in the title or the abstract | | P1, line 4 |
|  |  | (*b*) Provide in the abstract an informative and balanced summary of what was done and what was found | | P1 |
| Introduction | | | |  |
| Background/rationale | 2 | Explain the scientific background and rationale for the investigation being reported | | P2 -3 |
| Objectives | 3 | State specific objectives, including any prespecified hypotheses | | P3, lines 16-27 |
| Methods | | | |  |
| Study design | 4 | Present key elements of study design early in the paper | | P3-5  Figure1 and 2 |
| Setting | 5 | Describe the setting, locations, and relevant dates, including periods of recruitment, exposure, follow-up, and data collection | | P3-5 |
| Participants | 6 | (*a*) Give the eligibility criteria, and the sources and methods of selection of participants | | P7, lines 12-23 |
| Variables | 7 | Clearly define all outcomes, exposures, predictors, potential confounders, and effect modifiers. Give diagnostic criteria, if applicable | | P3-5  Figure1 |
| Data sources/ measurement | 8* | For each variable of interest, give sources of data and details of methods of assessment (measurement). Describe comparability of assessment methods if there is more than one group | | P3-5 |
| Bias | 9 | Describe any efforts to address potential sources of bias | | P3-5 |
| Study size | 10 | Explain how the study size was arrived at | | P3-5 |
| Quantitative variables | 11 | Explain how quantitative variables were handled in the analyses. If applicable, describe which groupings were chosen and why | | P3-5 |
| Statistical methods | 12 | (*a*) Describe all statistical methods, including those used to control for confounding | | P5 |
|  |  | (*b*) Describe any methods used to examine subgroups and interactions | | - |
|  |  | (*c*) Explain how missing data were addressed | | - |
|  |  | (*d*) If applicable, describe analytical methods taking account of sampling strategy | | - |
|  |  | (*e*) Describe any sensitivity analyses | | - |
| Results | | | |  |
| Participants | 13* | (a) Report numbers of individuals at each stage of study—eg numbers potentially eligible, examined for eligibility, confirmed eligible, included in the study, completing follow-up, and analysed | | P5,6  Figure 1 |
|  |  | (b) Give reasons for non-participation at each stage | | - |
|  |  | (c) Consider use of a flow diagram | | Figure 1 |
| Descriptive data | 14* | (a) Give characteristics of study participants (eg demographic, clinical, social) and information on exposures and potential confounders | | P5  Appendix Table 1 |
|  |  | (b) Indicate number of participants with missing data for each variable of interest | | - |
| Outcome data | 15* | Report numbers of outcome events or summary measures | | P 5,6 |
| Main results | 16 | (*a*) Give unadjusted estimates and, if applicable, confounder-adjusted estimates and their precision (eg, 95% confidence interval). Make clear which confounders were adjusted for and why they were included | P5,6  supplementary file (appendix tables 2-5)  Figure 4 | |
|  |  | (*b*) Report category boundaries when continuous variables were categorized | | - |
|  |  | (*c*) If relevant, consider translating estimates of relative risk into absolute risk for a meaningful time period | | - |
| Other analyses | 17 | Report other analyses done—eg analyses of subgroups and interactions, and sensitivity analyses | | - |
| Discussion | | | |  |
| Key results | 18 | Summarise key results with reference to study objectives | | P 6-8 |
| Limitations | 19 | Discuss limitations of the study, taking into account sources of potential bias or imprecision. Discuss both direction and magnitude of any potential bias | | P 8, lines 15-23 |
| Interpretation | 20 | Give a cautious overall interpretation of results considering objectives, limitations, multiplicity of analyses, results from similar studies, and other relevant evidence | | P 6-8 |
| Generalisability | 21 | Discuss the generalisability (external validity) of the study results | | P 8, lines 15-23 |
| Other information | | | |  |
| Funding | 22 | Give the source of funding and the role of the funders for the present study and, if applicable, for the original study on which the present article is based | | P 9, lines 18-20 |

*Give information separately for exposed and unexposed groups.

**Note:** An Explanation and Elaboration article discusses each checklist item and gives methodological background and published examples of transparent reporting. The STROBE checklist is best used in conjunction with this article (freely available on the Web sites of PLoS Medicine at http://www.plosmedicine.org/, Annals of Internal Medicine at http://www.annals.org/, and Epidemiology at http://www.epidem.com/). Information on the STROBE Initiative is available at [www.strobe-statement.org](http://www.strobe-statement.org).

**CLAIM Checklist**

| Section / Topic | No. | Item |  |
| --- | --- | --- | --- |
| TITLE / ABSTRACT |  |  | **Page** |
|  | **1** | Identification as a study of AI methodology, specifying the category of technology used (e.g., deep learning) | **1** |
|  | **2** | Structured summary of study design, methods, results, and conclusions | **1** |
| INTRODUCTION |  |  |  |
|  | **3** | Scientific and clinical background, including the intended use and clinical role of the AI approach | **2-3** |
|  | **4** | Study objectives and hypotheses | **2-3** |
| METHODS |  |  |  |
| *Study Design* | **5** | Prospective or retrospective study | **3-5** |
|  | **6** | Study goal, such as model creation, exploratory study, feasibility study, non-inferiority trial | **3-5** |
| *Data* | **7** | Data sources | **3-5** |
|  | **8** | Eligibility criteria: how, where, and when potentially eligible participants or studies were identified (e.g., symptoms, results from previous tests, inclusion in registry, patient-care setting, location, dates) | **3-5** |
|  | **9** | Data pre-processing steps | **3-5** |
|  | **10** | Selection of data subsets, if applicable | **3-5** |
|  | **11** | Definitions of data elements, with references to Common Data Elements | **3-5** |
|  | **12** | De-identification methods | **3-5** |
|  | **13** | How missing data were handled | **-** |
| *Ground Truth* | **14** | Definition of ground truth reference standard, in sufficient detail to allow replication | **3-5** |
|  | **15** | Rationale for choosing the reference standard (if alternatives exist) | **3-5** |
|  | **16** | Source of ground-truth annotations; qualifications and preparation of annotators | **3-5** |
|  | **17** | Annotation tools | **3-5** |
|  | **18** | Measurement of inter- and intrarater variability; methods to mitigate variability and/or resolve discrepancies | **3-5** |
| *Data Partitions* | **19** | Intended sample size and how it was determined | **3-5** |
|  | **20** | How data were assigned to partitions; specify proportions | **3-5** |
|  | **21** | Level at which partitions are disjoint (e.g., image, study, patient, institution) | **3-5** |
| *Model* | **22** | Detailed description of model, including inputs, outputs, all intermediate layers and connections | **3-5** |
|  | **23** | Software libraries, frameworks, and packages | **3-5** |
|  | **24** | Initialization of model parameters (e.g., randomization, transfer learning) | **3-5** |
| *Training* | **25** | Details of training approach, including data augmentation, hyperparameters, number of models trained | **3-5** |
|  | **26** | Method of selecting the final model | **3-5** |
|  | **27** | Ensembling techniques, if applicable | **-** |
| *Evaluation* | **28** | Metrics of model performance | **3-5** |
|  | **29** | Statistical measures of significance and uncertainty (e.g., confidence intervals) | **3-5** |
|  | **30** | Robustness or sensitivity analysis | **-** |
|  | **31** | Methods for explainability or interpretability (e.g., saliency maps), and how they were validated | **-** |
|  | **32** | Validation or testing on external data | **3-5** |
| RESULTS |  |  |  |
| *Data* | **33** | Flow of participants or cases, using a diagram to indicate inclusion and exclusion | **5-6** |
|  | **34** | Demographic and clinical characteristics of cases in each partition | **5-6** |
| *Model performance* | **35** | Performance metrics for optimal model(s) on all data partitions | **5-6** |
|  | **36** | Estimates of diagnostic accuracy and their precision (such as 95% confidence intervals) | **-** |
|  | **37** | Failure analysis of incorrectly classified cases | **-** |
| DISCUSSION |  |  |  |
|  | **38** | Study limitations, including potential bias, statistical uncertainty, and generalizability | **8** |
|  | **39** | Implications for practice, including the intended use and/or clinical role | **6-8** |
| OTHER INFORMATION |  |  |  |
|  | **40** | Registration number and name of registry | **3** |
|  | **41** | Where the full study protocol can be accessed | **3** |
|  | **42** | Sources of funding and other support; role of funders | **9** |

**
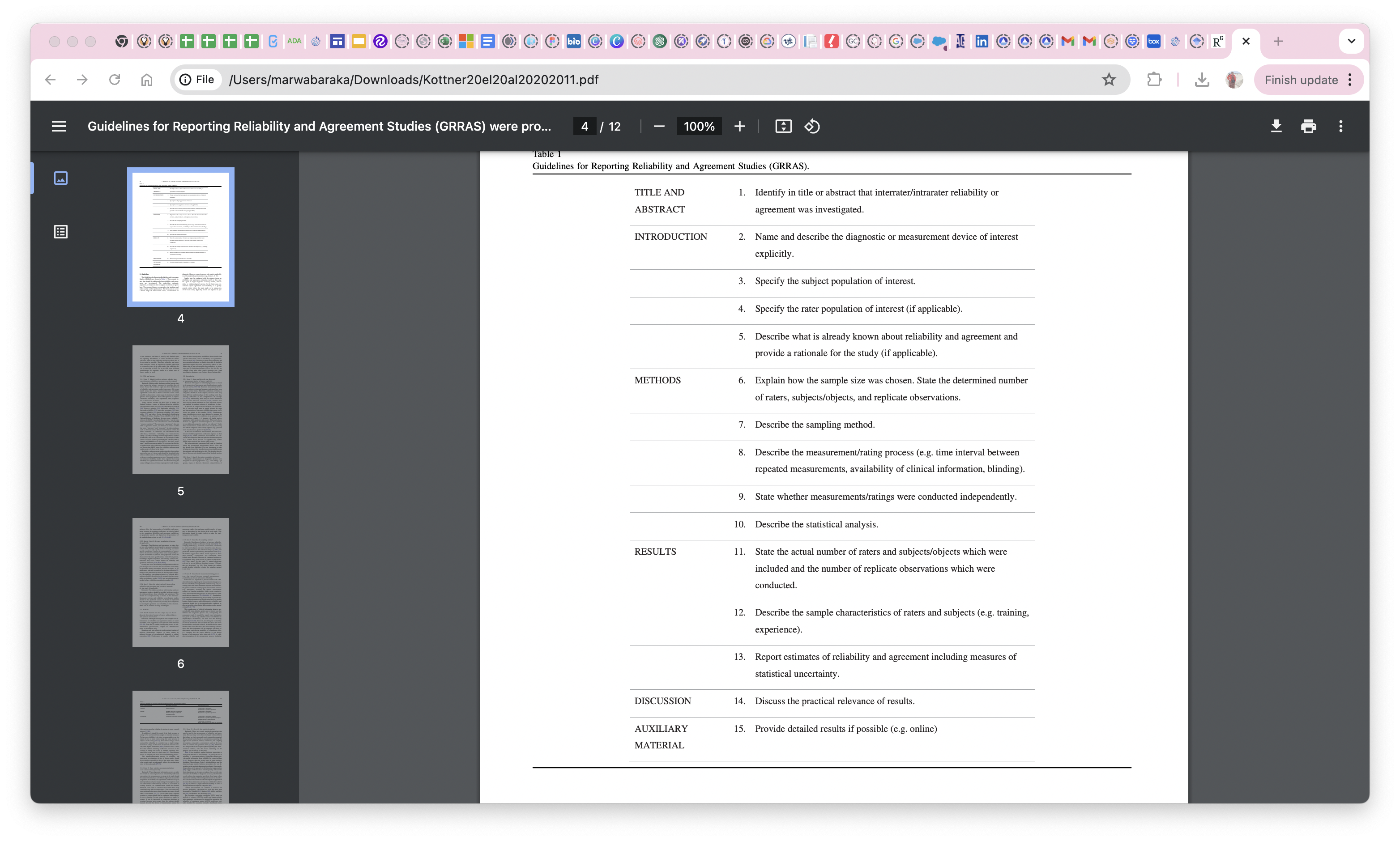
**

Page 2-3

Page 1

(Kottner et al. 2011)

Page 2-3

Page 2-3

Page 2-3

Page 3-5

Page 3-5

Page 3-5

Page 3-5

Page 5

Page 5-6

Page 5-6

Page 6-8

Page 5-6

All raw data and detailed results are available online and included in a supplementary file

**Regenerative Endodontic Procedure**

REPs were carried out for all patients by two expert endodontists (N.E. and P.S.).^1,2^ In the first appointment, following rubber dam isolation and access cavity preparations, in the immature teeth: minimal instrumentation was done to the working length using 2% taper stainless-steel hand file matching the size of the canal. For the mature teeth: the canals were instrumented by Pro-Taper Next (PTN) (Dentsply Sirona, York, PA) till sizes X5. This was done under copious irrigation with 20ml 1.5 % sodium hypochlorite (NaOCl) using 30-gauge side vented needle (Morris Dental Company Ltd. Dublin). Canals were dried then followed by Ultracal XS calcium hydroxide (Ultradent Products, GmbH, Germany) placement, 2mm short of the radiographic apex, according to the manufacturer's instructions.

At the second appointment (2 weeks after the 1^st^ visit), all patients were asymptomatic. Local anesthesia using 3% Mepivacaine without vasoconstrictor (Scandonset, Septodont, France) was administered followed by rubber dam isolation. Under the use of the endodontic microscope (Karl Kaps, GmbH & Co.KG, Germany), calcium hydroxide was removed by using 20 ml 17% EDTA (Prevest Direct, Jammu, India) followed by drying the canals using paper points. Induction of bleeding was done by over instrumenting by rotating a pre-curved K file # 25 (Dentsply Sirona, York, PA) at 2-3 mm beyond the radiographic apex. The blood was left in the canals for a few minutes to allow clotting, afterwards, Biodentine (Septodont, France) was carefully mixed and placed over the clot according to the manufacturer's instructions. The position and the quality of the cervical plug were assessed radiographically before proceeding with the final coronal restoration using light-cured resin-modiﬁed glass ionomer restorative material as a base (Riva, SD, Australia) and composite resin restoration (Nexcomp, Metabiomed, South Korea). High points were checked carefully to avoid traumatic occlusion. ^3^

Cone-beam computed tomographic images were taken at baseline and after 12 months using Veraviewepocs 3D R 100 (J Morita Corp, Koyoto, Japan) operating at 90 kV and 8mA with an exposure time of 9.4 seconds, field of view of 40 x 40, 100×80mm,80×80mm and voxel size of 0.125mm.

**Hardware and software specifications, and code/data sharing information**

**Hardware and software specifications**

| Hardware Component | Specification |
| --- | --- |
| GPU | Nvidia 4090-RTX 24GB |
| CPU | Intel i7 13700K - 24 core |
| RAM | 32 GB DDR4 |
| SSD | 1TB M2 |
| Parameter | Value |
| Framework | PyTorch and TorchIO |
| Optimizer | SGD (lr: 0.01) / Decay using Plateau of 20 epochs |
| Loss Function | Jaccard Loss |
| Volume Patching | 16x16x16 (using LabelSampler) |
| Gradient accumulation | Every 8 epochs |

**Code/data sharing information:**

The data used to train our network is publicly available as [Toothfairy](https://ditto.ing.unimore.it/toothfairy/?fbclid=IwAR2gRzMOyfsWnRUgscWV1Bhx1BW2BqhUsJs5s66vPH2cai0Cf_DqiftLzyw) [[link](https://ditto.ing.unimore.it/toothfairy/?fbclid=IwAR2gRzMOyfsWnRUgscWV1Bhx1BW2BqhUsJs5s66vPH2cai0Cf_DqiftLzyw)]. The dataset are stored as a password protected zipped file in the Alexandria University repository; <http://eng.staff.alexu.edu.eg/staff/mtorki/Research/Data/JDR_Files/>. The code is available in a private repository on Github <https://github.com/mahmoudgamal0/Pulpy3D-Seed> as well as a password protected zipped file in the Alexandria University repository, <http://eng.staff.alexu.edu.eg/staff/mtorki/Research/Data/JDR_Files/>.


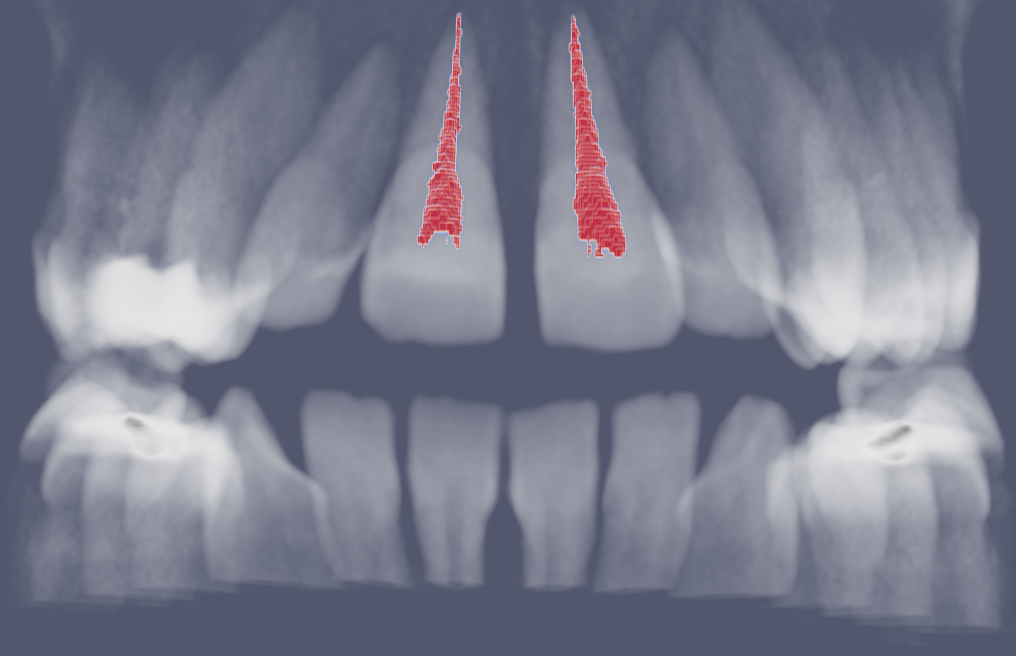

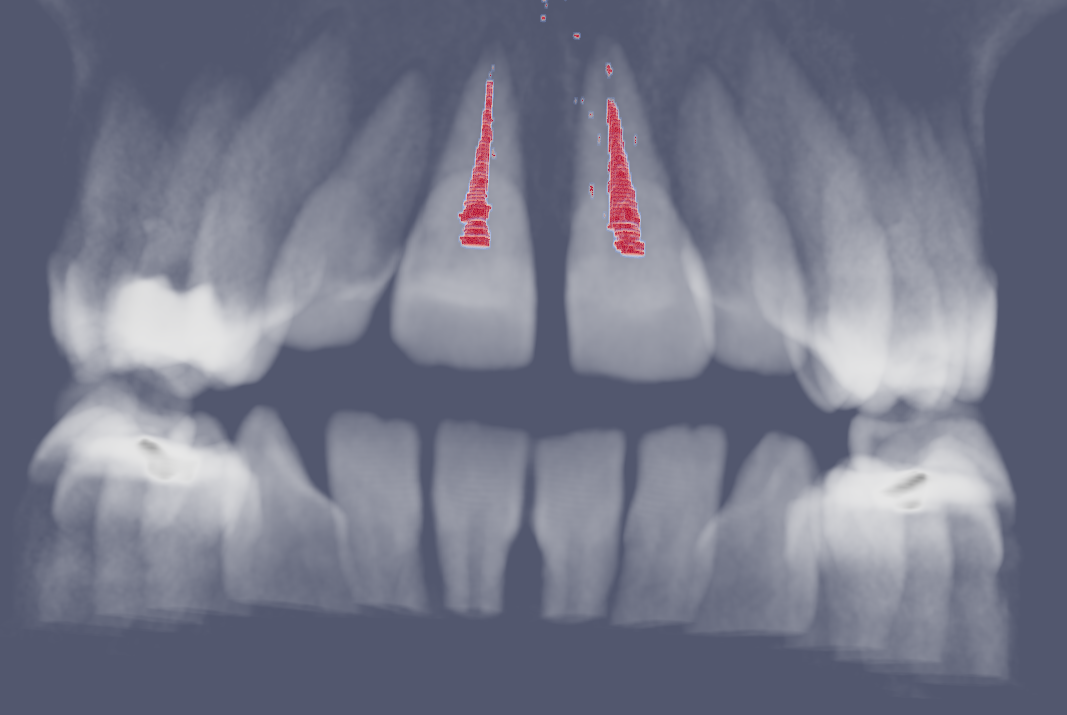


Before Treatment (Semiautomated)

Before Treatment (Automated)


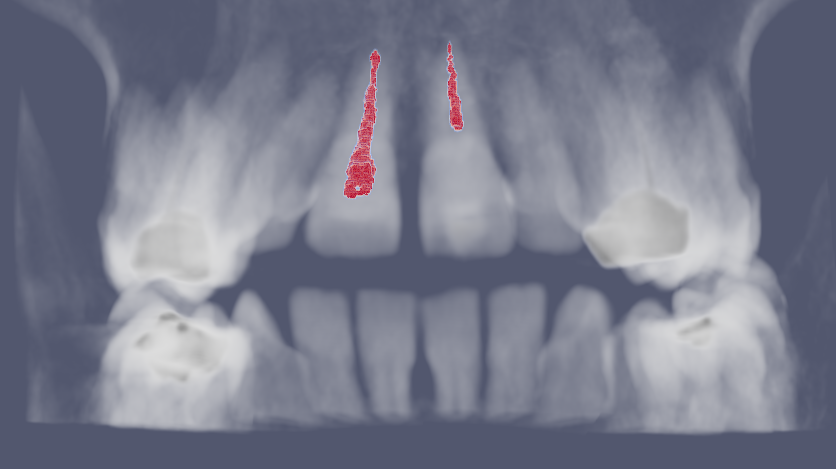

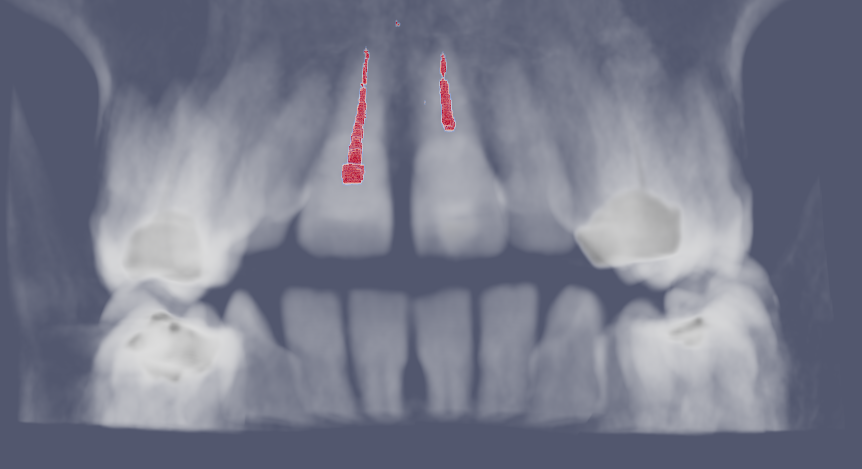


After Treatment (Semiautomated)

After Treatment (Automated)

Appendix Figure 1: Case 1


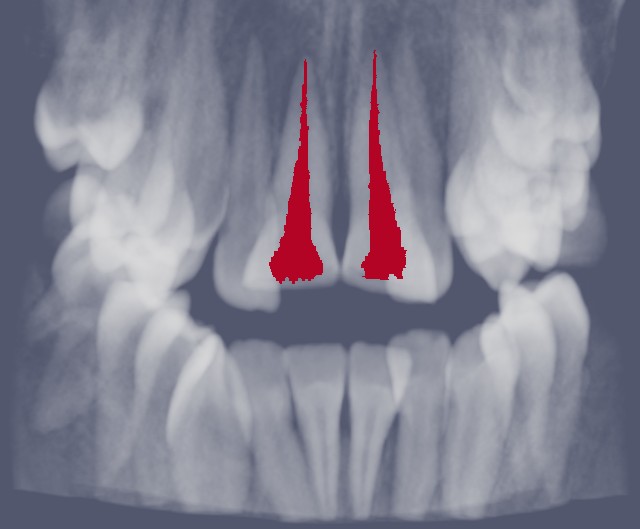

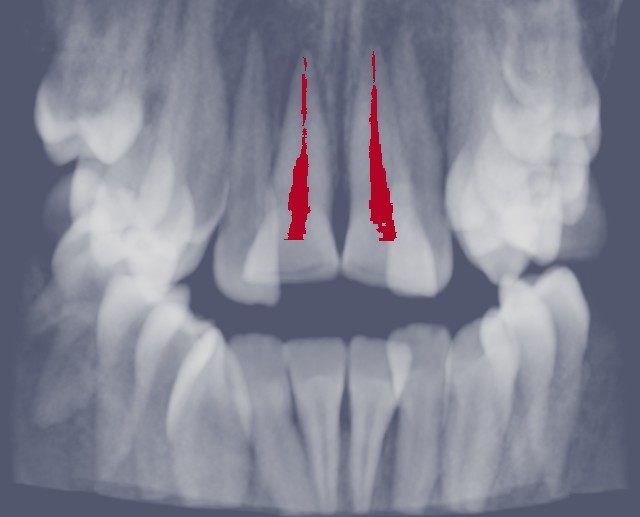


Before Treatment (Semiautomated)

Before Treatment (Automated)


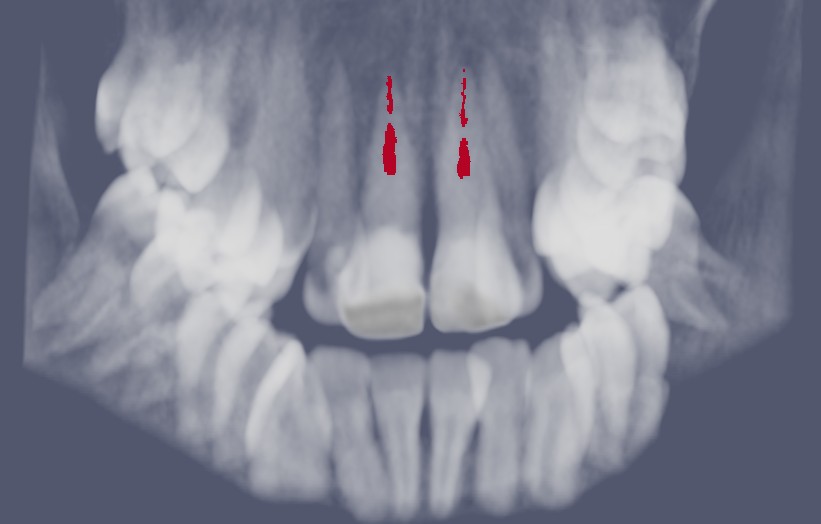

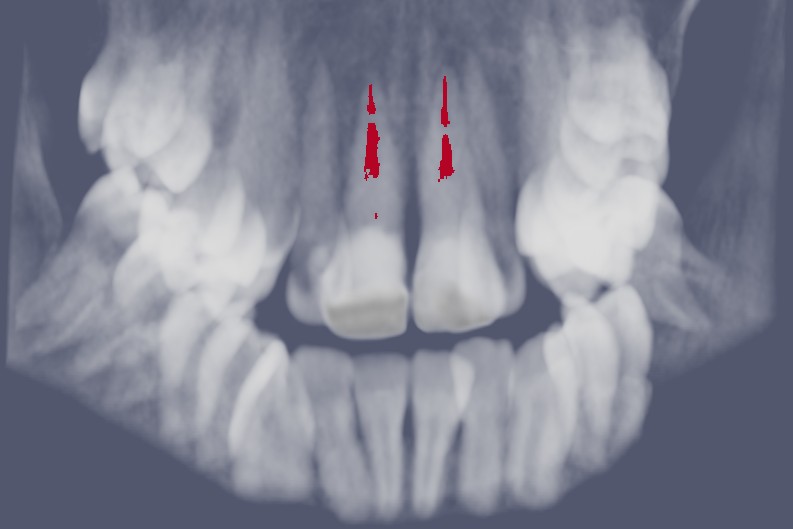


After Treatment (Semiautomated)

After Treatment (Automated)

Appendix Figure 2: Case 2


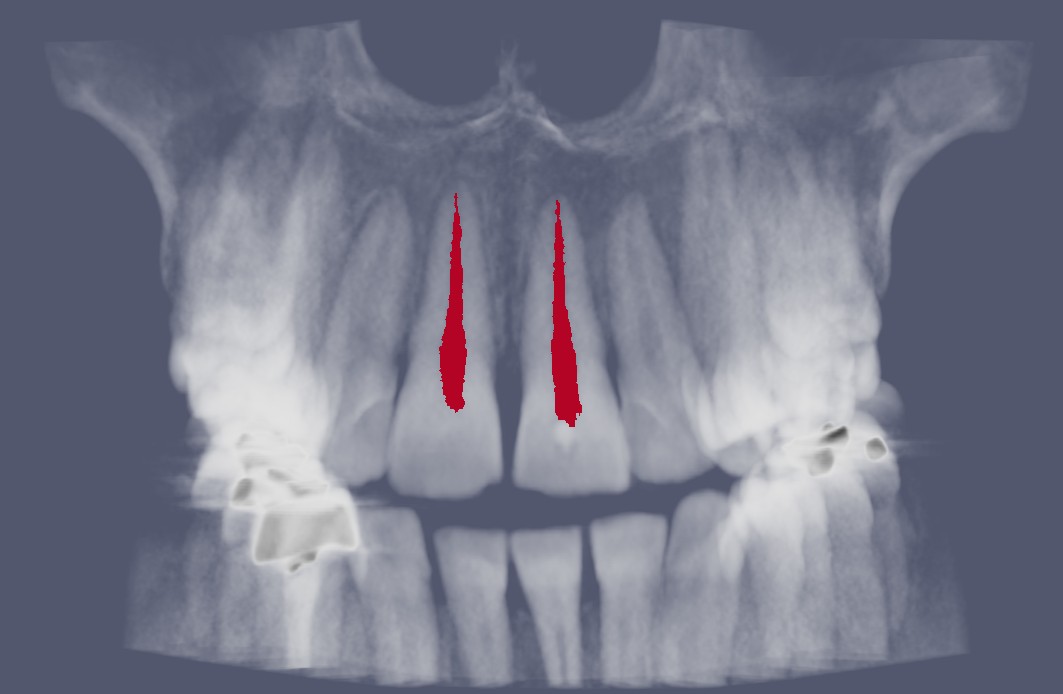

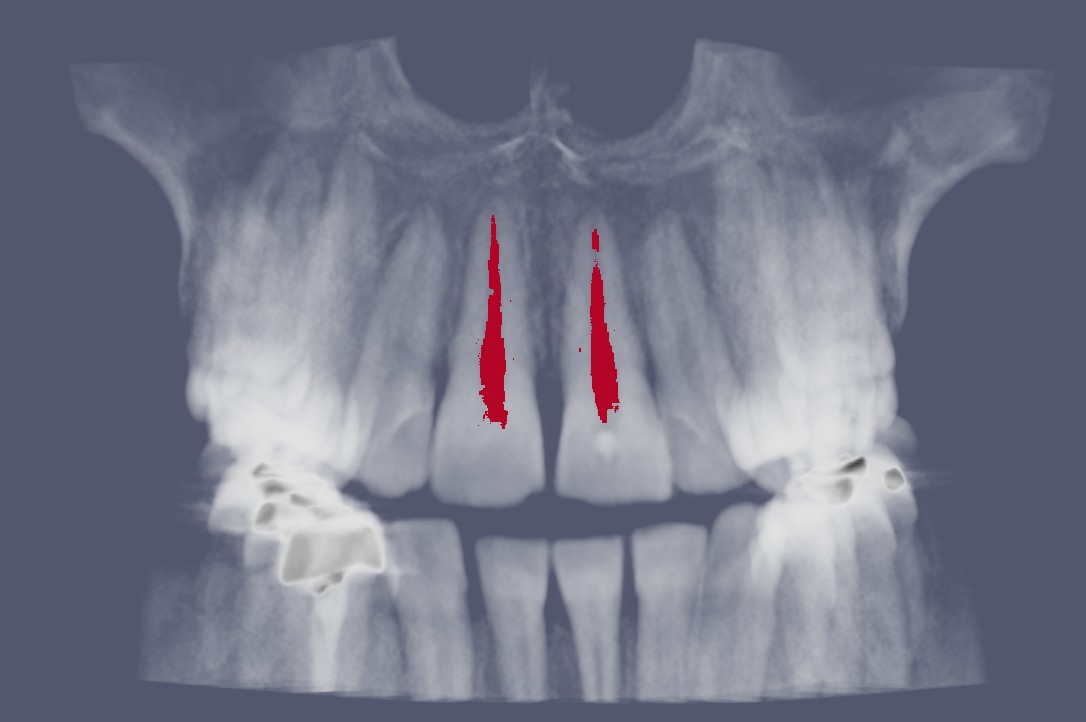


Before Treatment (Semiautomated)

Before Treatment (Automated)


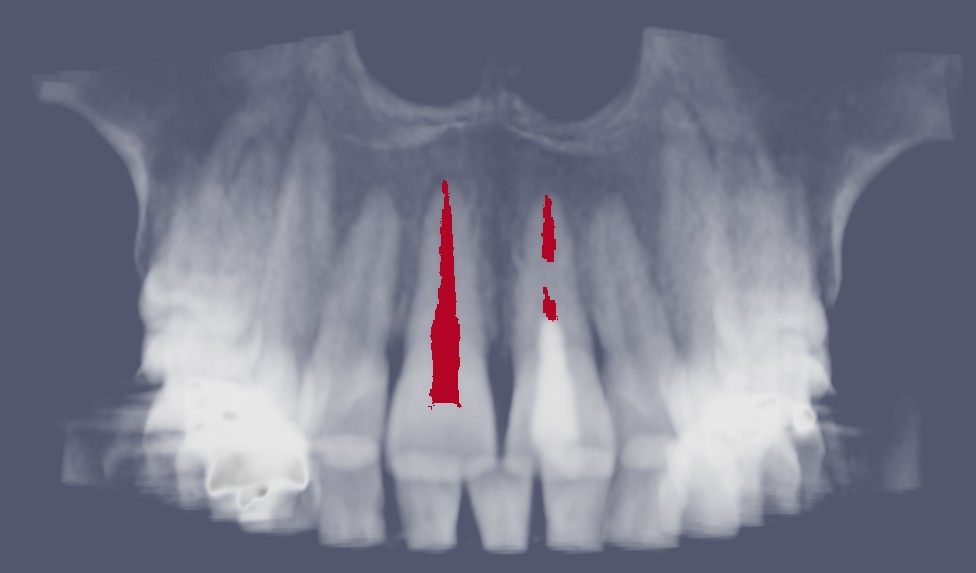

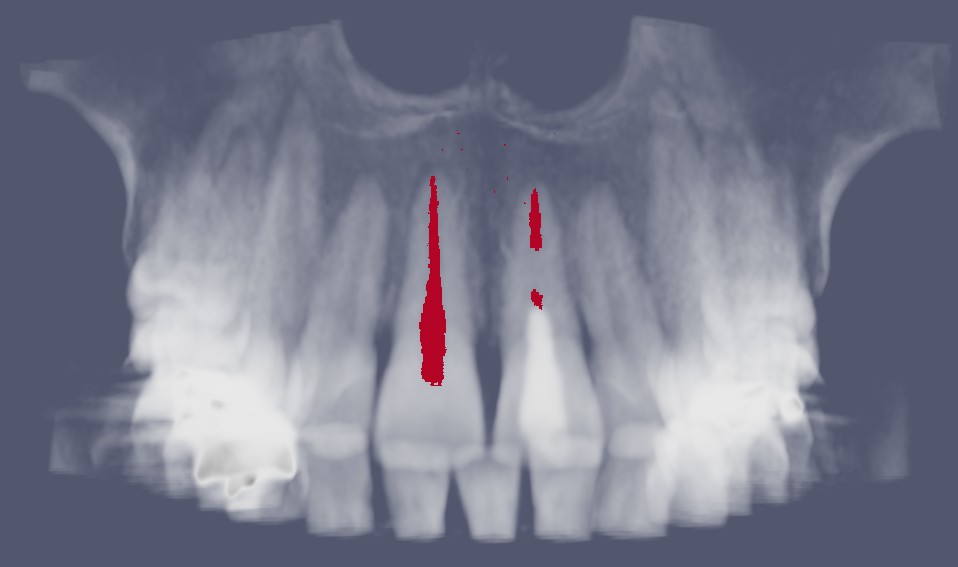


After Treatment (Semiautomated)

After Treatment (Automated)

Appendix Figure 3: Case 3


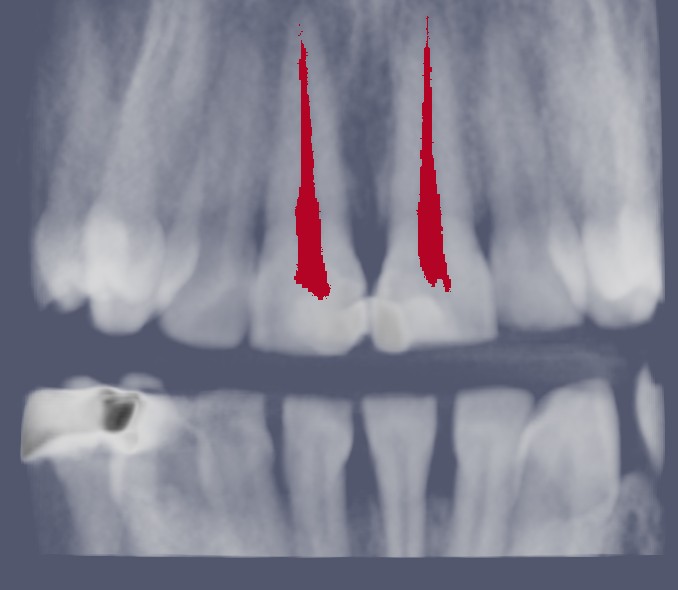

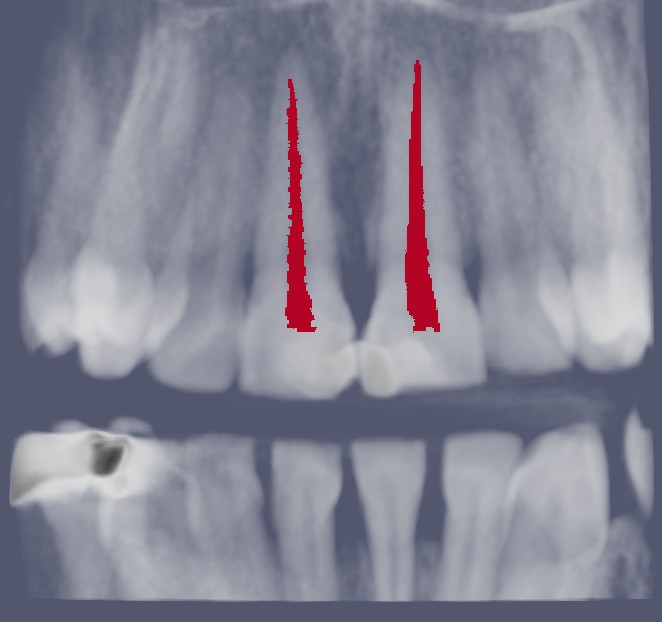


Before Treatment (Semiautomated)

Before Treatment (Automated


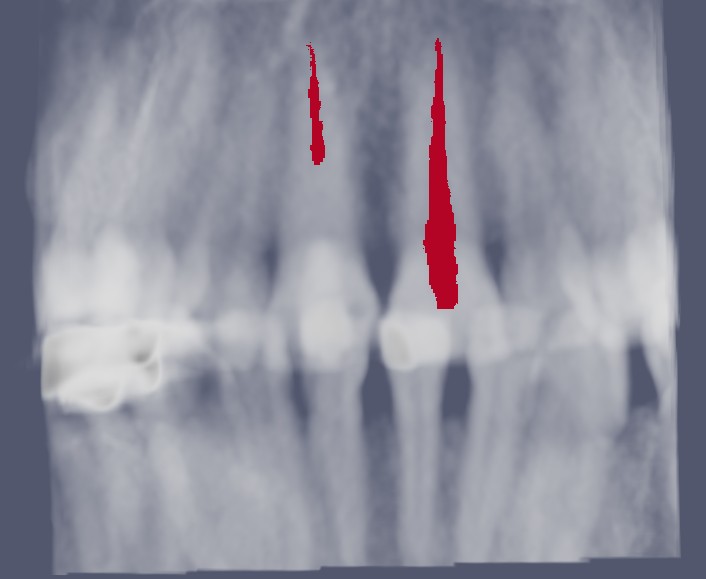

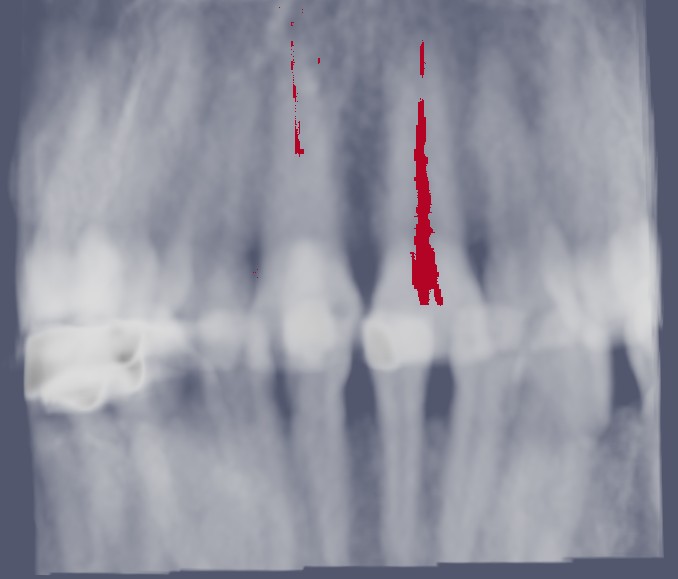


After Treatment (Semiautomated)

After Treatment (Automated)

Appendix Figure 4: Case 4


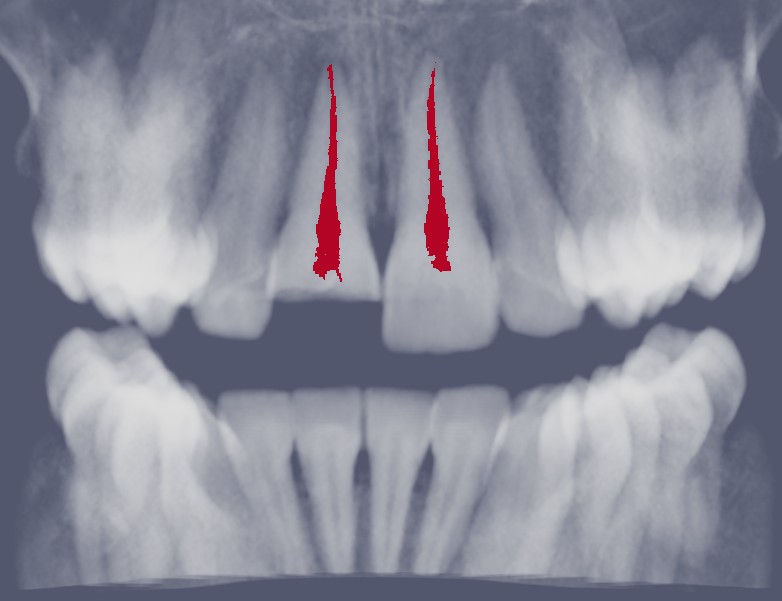

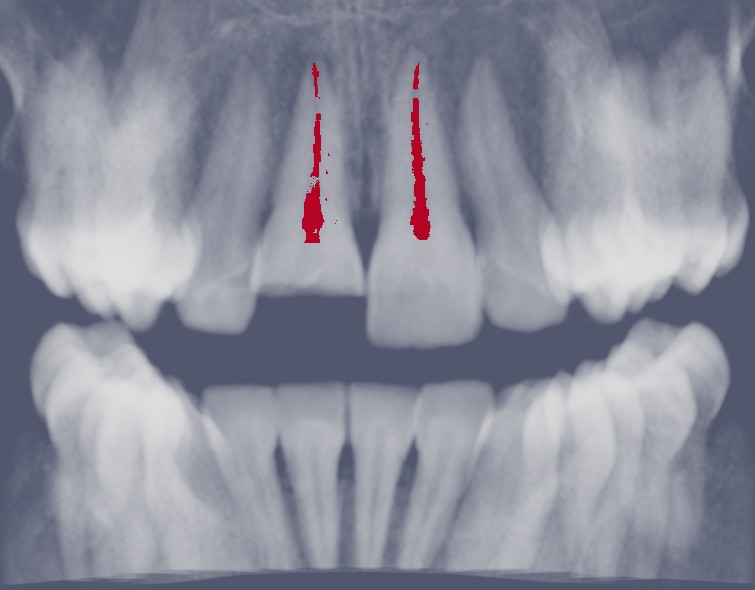


Before Treatment (Semiautomated)

Before Treatment (Automated)


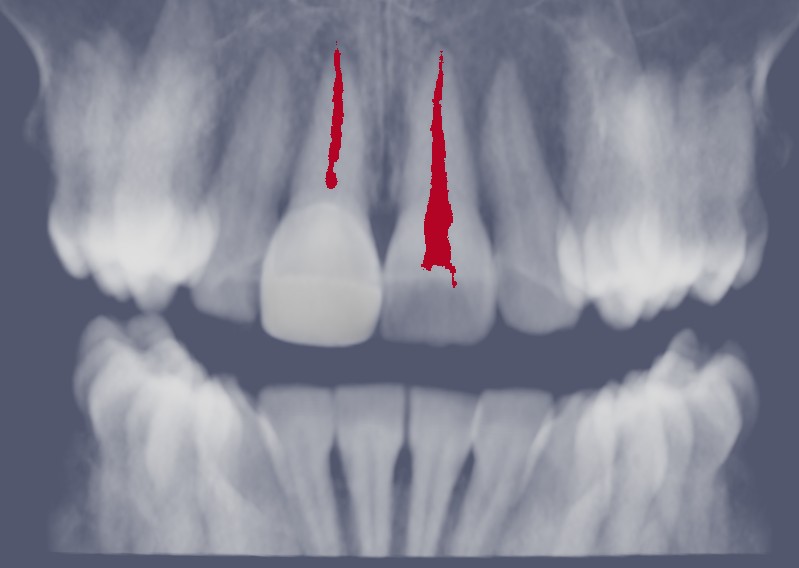

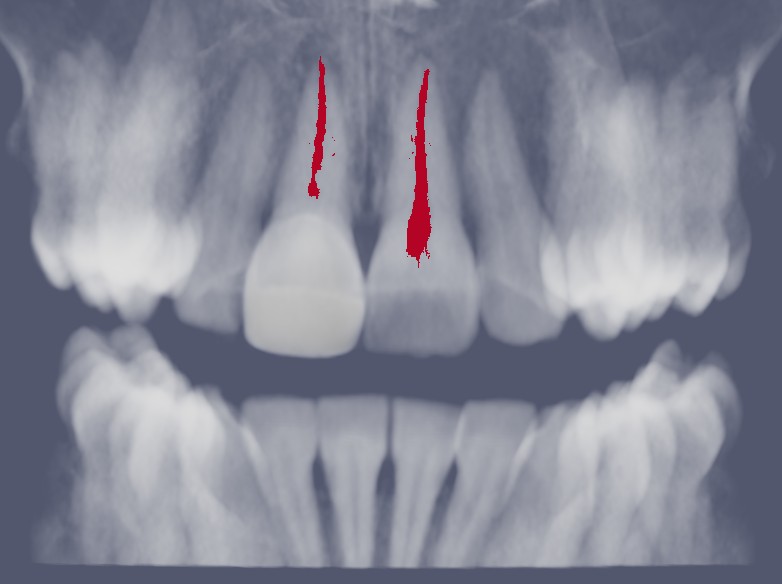


After Treatment (Semiautomated)

Appendix Figure 5: Case 5

After Treatment (Automated)


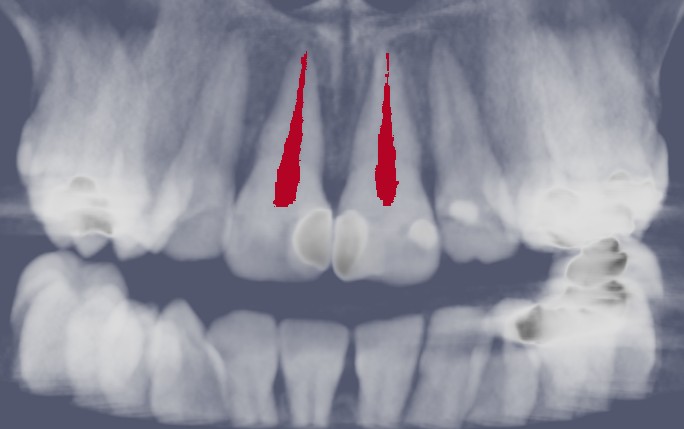

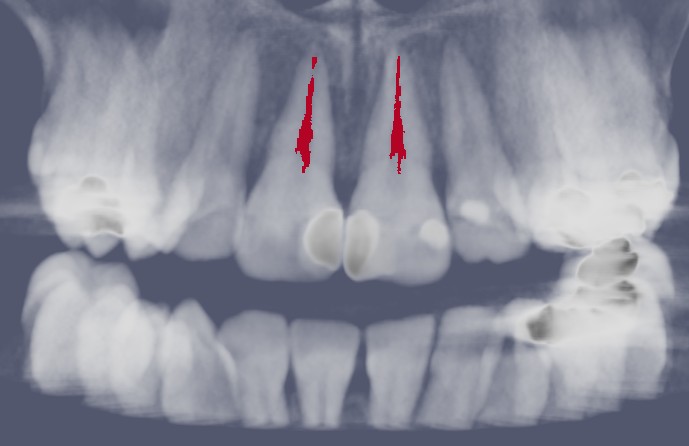


Before Treatment (Semiautomated)

Before Treatment (Automated)


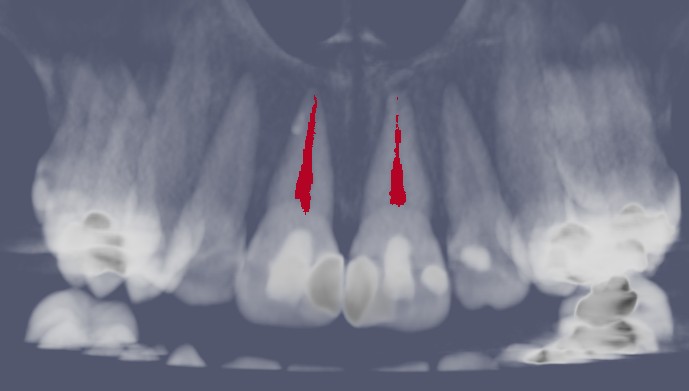


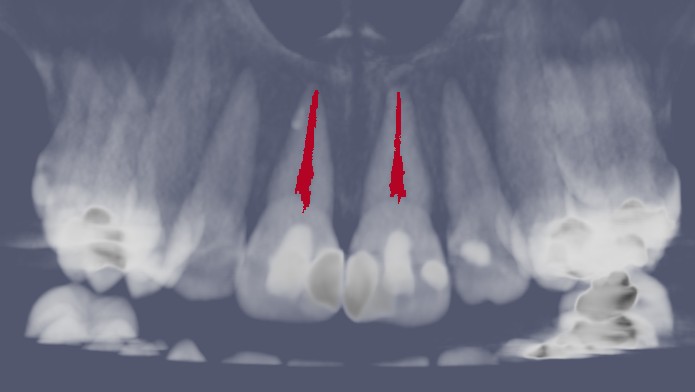


After Treatment (Semiautomated) After Treatment (Automated)

Appendix Figure 6: Case 6

**Appendix Table 1: Demographics and clinical variables by tooth maturity status in a cohort of sixteen patients with REPs (n=23)**

| **Variables** | | **Overall (n = 23)** | **Tooth maturity (n = 23)** | | ***P* value** |
| --- | --- | --- | --- | --- | --- |
|  |  |  | **Mature (n = 8)** | **Immature (n = 15)** |  |
| **Age in years** | **Median (IQR)** | 13 (10, 21) | 22 (20.25, 34.75) | 10 (9, 13) | < 0.001**^a^***** |
|  | **Mean ± SD** | 16.09 ± 8.85 | 26.25 ± 7.54 | 10.67 ± 2.06 |  |
| **Gender** | **Males n (%)** | 13 (56.52) | 3 (37.50) | 10 (66.67) | 0.18**^b^** |
|  | **Females n (%)** | 10 (43.48) | 5 (62.50) | 5 (33.33) |  |
| **Diagnosis** | **Pulp necrosis with asymptomatic apical periodontitis n (%)** | 14 (60.87) | 6 (75) | 8 (53.34) | 0.40**^c^** |
|  | **Pulp necrosis with chronic apical abscess n (%)** | 9 (39.13) | 2 (25) | 7 (46.67) |  |
| **Etiology** | **Trauma n (%)** | 17 (73.9) | 2 (25) | 15 (100) | < 0.001**^c^***** |
|  | **Caries/ Defective Restorations n (%)** | 6 (26.1) | 6 (75) | 0 (0) |  |

*SD* Standard deviation, *IQR* interquartile range, ^a^Mann-Whitney U test, ^b^Chi square test, ^c^Fisher’s exact

***Significant at *p* < 0.001

An equal number of CON teeth was analysed for each group.

**Appendix Table 2: Difference in root volume changes between teeth treated with regenerative endodontic procedures (REP) and their contralaterals (CON) (n = 23)**

| **Type of teeth** | | **REP**  **(mm^3^)** | **CON**  **(mm^3^)** | **95% CI** | ***p* value** |
| --- | --- | --- | --- | --- | --- |
| **Mature** (n= 8) | **Median (IQR)** | 7.40 (2.62, 12.08) | 9.65 (7.63, 12.55) | (-10.42, 11.41) | 0.48 ^a^ |
|  | **Mean ± SD** | 10.26 ± 11.83 | 9.77 ± 2.53 |  |  |
| **Immature** (n= 15) | **Median (IQR)** | 11.90 (7.60, 34.70) | 13.70 (2.60, 24.10) | (-13.33, 12.73) | 0.78 ^a^ |
|  | **Mean ± SD** | 20.47 ± 23.88 | 20.77 ± 26.21 |  |  |
| ***p* value** | | 0.13 ^b^ | 0.51 ^b^ | - | - |
| **95% CI** | | (-8.60, 29.02) | (-8.53, 30.53) |  |  |

*IQR* interquartile range, *SD* standard deviation*, CI* confidence interval, ^a^Wilcoxon signed-rank test, ^b^Mann-Whitney U test

An equal number of CON teeth was analysed for each group.

**Appendix Table 3: Difference in volumetric dentinal wall changes between teeth treated with regenerative endodontic procedures (REP) and their contralateral (CON) (n = 23)**

| **Type of teeth** | | **REP**  **(mm^3^)** | **CON**  **(mm^3^)** | **95% CI** | ***p* value** |
| --- | --- | --- | --- | --- | --- |
| **Mature** (n= 8) | **Median (IQR)** | 6.86 (1.44, 11.46) | 9.65 (7.63, 12.55) | (-10.99, 9.84) | 0.40 ^a^ |
|  | **Mean ± SD** | 9.19 ± 11.15 | 9.77 ± 2.53 |  |  |
| **Immature** (n= 15) | **Median (IQR)** | 11.60 (5.00, 23.46) | 13.70 (2.60, 24.10) | (-17.60, 9.95) | 0.95 ^a^ |
|  | **Mean ± SD** | 16.95 ± 20.95 | 20.77 ± 26.21 |  |  |
| ***p* value** | | 0.29 ^b^ | 0.51 ^b^ | - | - |
| **95% CI** | | (-8.89, 24.39) | (-8.53, 30.53) |  |  |

*IQR* interquartile range, *SD* standard deviation*, CI* confidence interval, ^a^Wilcoxon signed-rank test, ^b^Mann-Whitney U test

An equal number of CON teeth was analysed for each group.

**Appendix Table 4: Difference in intracanal calcification between mature and immature teeth with regenerative endodontic procedures (REP) (n = 23)**

| **Type of teeth** | | **Mature REP**  (n = 8) | **Immature REP**  (n = 15) | **95% CI** | p value |
| --- | --- | --- | --- | --- | --- |
| **Intracanal calcification (mm^3^)** | **Median (IQR)** | 0.61 (0.47, 1.44) | 2.60 (0.00, 6.29) | (-5.48, 0.08) | 0.68 |
|  | **Mean ± SD** | 1.03 ± 0.91 | 3.72 ± 4.92 |  |  |
| **Calcific Bridge (mm)** | **Location from RA** | 4.34 (1.997, 7.799 | 4.95 (2.48-9.43) (n=5) | - | |

*IQR* interquartile range, *SD* standard deviation*, CI* confidence interval, Mann-Whitney u test

**Appendix Table 5: Difference in root length changes between teeth treated with regenerative endodontic procedures (REP) and their contralateral (CON) (n = 23)**

| **Type of teeth** | | **REP**  **(mm)** | **CON**  **(mm)** | **95% CI** | ***p* value** |
| --- | --- | --- | --- | --- | --- |
| **Mature** (n= 8) | **Median (IQR)** | 0.03 (-0.01, 0.30) | 0.10 (0.02, 0.16) | (-1.20, 0.20) | 0.26 ^a^ |
|  | **Mean ± SD** | 0.09 ± 0.19 | 0.10 ± 0.08 |  |  |
| **Immature** (n= 15) | **Median (IQR)** | 0.33 (0.13, 0.6) | 0.19 (0.02, 0.82) | (-1.46, 1.02) | 0.53 ^a^ |
|  | **Mean ± SD** | 0.52 ± 0.89 | 0.77 ± 1.29 |  |  |
| ***p* value** | | 0.04* ^b^ | 0.19 ^b^ | - | - |
| **95% CI** | | (0.06, 2.50) | (-1.38, 0.04) |  |  |

*IQR* interquartile range, *SD* standard deviation, *CI* confidence interval, ^a^Wilcoxon signed-rank test, ^b^Mann-Whitney U test

*Statistically significant at *p* < 0.05.

An equal number of CON teeth was analysed for each group.

**Appendix Table 6: Difference in volumetric pulp changes between** **teeth treated with regenerative endodontic procedures (REP) and their contralateral (CON) (n = 23)**

| **Type of teeth** | | **REP**  **(mm^3^)** | **CON**  **(mm^3^)** | **95% CI** | ***p* value** |
| --- | --- | --- | --- | --- | --- |
| **Mature** (n= 8) | **Median (IQR)** | -4.86 (-5.46, -2.20) | -1.34 (-2.82, -0.35) | (-4.59, -0.14) | 0.05 *^a^ |
|  | **Mean ± SD** | -3.92 ± 1.84 | -1.56 ± 1.18 |  |  |
| **Immature** (n= 15) | **Median (IQR)** | -3.20 (-7.48, -1.12) | -6.44 (-18.42, -4.94) | (-1.39, 8.51) | 0.12 ^a^ |
|  | **Mean ± SD** | -7.43 ± 9.47 | -10.99 ± 8.87 |  |  |
| ***p* value** | | 0.87 ^b^ | <0.001 ^b^ | - | - |
| **95% CI** | | (-8.88, 1.86) | (-14.39, -4.47) |  |  |

*IQR* interquartile range, *SD* standard deviation, *CI* confidence interval, ^a^Wilcoxon signed-rank test, ^b^Mann-Whitney U test

*Statistically significant at *p* < 0.05.

An equal number of CON teeth was analysed for each group.

**Appendix Table 7: Difference between semiautomated and automated pulpal segmentation (3D UNet) in measuring** **volumetric pulpal changes in mature teeth treated with regenerative endodontic procedures (REP) (n=8) and their contralateral (CON) (n = 12)**

| **Tooth** | | **3D UNet**  **(mm^3^)** | **Semiautomated**  **(mm^3^)** | **95% CI** | ***p* value** |
| --- | --- | --- | --- | --- | --- |
| **REP** (n=8) | **Median (IQR)** | -4.91 (-5.36, -2.60) | -4.86 (-5.46, -2.20) | (-1.05, 0.35) | 0.26 |
|  | **Mean ± SD** | -4.28 ± 1.44 | -3.92 ± 1.84 |  |  |
| **CON** (n=12) | **Median (IQR)** | -1.67 (-3.97, -0.64) | -2.35 (-3.03, -1.69) | (-0.55, 0.72) | 0.69 |
|  | **Mean ± SD** | -2.14 ± 1.65 | -2.23 ± 1.04 |  |  |

*IQR* interquartile range, *SD* standard deviation, *CI* confidence interval. Wilcoxon signed-rank test

*Statistically significant at *p* < 0.05.

ICC = 0.92, p = <0.001

**References**

*1. El-Kateb NM, El-Backly RN, Amin WM, Abdalla AM. Quantitative Assessment of Intracanal Regenerated Tissues after Regenerative Endodontic Procedures in Mature Teeth Using Magnetic Resonance Imaging: A Randomized Controlled Clinical Trial. J Endod. 2020;46(5):563-574.*

*2. Sharaf PH, El Backly RM, Sherif RA, Zaazou AM, Hafez SF. Microbial identification from traumatized immature permanent teeth with periapical lesions using matrix-assisted laser desorption/ionization time-of-flight mass spectrometry. BMC Oral Health.2022;22(1):661.*

*3. Lee JY, Kersten DD, Mines P, Beltran TA. Regenerative endodontic procedures among endodontists: a web-based survey. J. Endod. 2018;44(2):250-255.*
